# Community-based screening and testing for Coronavirus in Cape Town, South Africa: Short report

**DOI:** 10.4102/phcfm.v12i1.2499

**Published:** 2020-06-03

**Authors:** Neal David, Robert Mash

**Affiliations:** 1 Metropolitan Health Services, Western Cape Department of Health, Cape Town, South Africa; 2 Division of Family Medicine and Primary Care, Faculty of Medicine and Health Sciences, Stellenbosch University, Cape Town, South Africa

**Keywords:** primary care, COVID-19, community health workers, mass screening, community-orientated primary care

## Abstract

Corona Virus Infectious Disease 2019 (COVID-19) was first reported in Cape Town in March 2020 and the transmission was soon observed in local communities. Cape Town has many vulnerable communities because of poverty, overcrowding and comorbidities, although it has a relatively small elderly population. Amongst the unique and early responses to the pandemic in South Africa has been the strategy of community screening and testing (CST). This process has been drawn from health department’s prior adoption of a community-orientated primary care (COPC) approach, which relies on teams of community health workers working in delineated communities to prevent disease and provide early interventions for those at higher risk. The COPC principles were applied in the CST programme, which involved collaboration between facility and community-based teams, linking public health and primary care approaches, careful mapping of cases in highly vulnerable communities, targeted screening around cases, testing of those that screened positive, health education and linkage to primary care. The overall aim was to slow down transmission through early identification and isolation of diagnosed cases. Key challenges involved the designing of a screening tool with appropriate sensitivity and specificity as well as the logistics of staffing, transport, consumables, data collection and capture, security, ablutions and personal protective equipment. Key opportunities included synergies between CST and evolving commitment to COPC in the health system. Key threats were the deteriorating security situation in the most vulnerable communities because of loss of income, food insecurity and CST distrust as well as increasing turn-around-times for test results.

## Introduction

The first case of Coronavirus infectious disease 2019 (COVID-19) in the Western Cape, South Africa, was reported on 11 March 2020. Initial cases were imported by travellers and largely reported in well-resourced areas of the Cape Town Metropole. However, transmission quickly took place amongst people with no travel history, and local cases soon outnumbered imported ones.

The National Department of Health quickly recommended community screening and testing (CST).^1^ There was an acceptance that the virus would ultimately penetrate South African society, but efforts taken to slow down transmission would provide health services enough time to prepare for its check.

Cape Town has a high number of poor, densely populated communities, where people live in informal settlements, backyard shacks and overcrowded houses. Although the proportion of elderly is small, there is a high proportion of population with comorbidities such as human immunodeficiency virus (HIV), tuberculosis (TB), hypertension, diabetes and chronic lung disease that put them more at risk of severe infection.

Community-orientated primary care (COPC) has been a cornerstone of South Africa’s policy to strengthen primary health care.^2^ In Cape Town, COPC was developed through community health worker (CHW) teams employed by local non-profit organisations (NPOs) under contract to the Department of Health. The metropolitan health services had 2500 CHWs at the beginning of the outbreak. Community health workers worked under the supervision of professional nurses and were responsible for delineated geographic areas linked to a local primary care facility.

## Community screening and testing

The Cape Town metro is divided into four sub-structures, each of which developed capacity for CST. In each sub-structure, a comprehensive programme manager created teams of people from the local primary care facilities and NPOs. A central co-ordinator (N.D.) provided strategic and logistic support.

**FIGURE 1:**
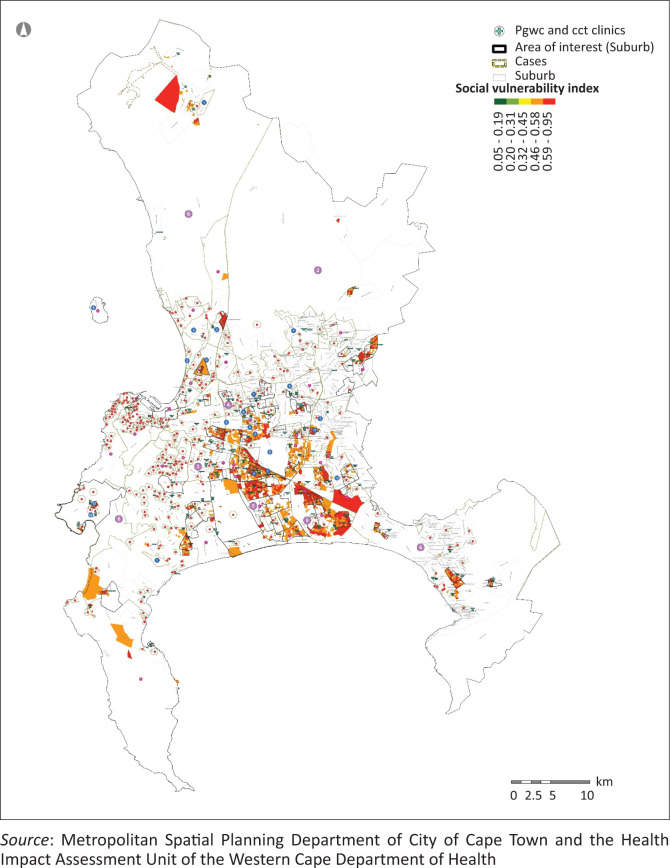
Cape Town Metropole illustrating the presence of COVID-19 cases and areas with a high social vulnerability index.

Community screening and testing was guided by the following principles: (1) presence of cases and (2) social vulnerability of community. Geographic information systems mapped known cases with the social vulnerability index (SVI) of their communities (Figure 1). These maps were used to target CST and define an area around approximately 200 households. This approach was preferred to general community screening as it was likely to yield more cases and be more efficient. The aim was to rapidly identify and isolate cases so as to prevent onward transmission.

Community health workers worked door to door in pairs and used a set of questions to determine whether any person had COVID-19 symptoms (Box 1). They wore masks and did not enter the premises. If someone was screened positive, then they were sent to a local mobile testing centre or primary care facility. Mobile testing centres included clinicians from local primary care facility, who collected naso- and oro-pharyngeal swabs, as well as administrative and support staff. The testing centre also acted as a base for coordinators and CHWs. People waiting to be tested maintained a distance of at least 1 m.

BOX 1 Community health worker screening questionnaire.

Have you experienced any one of the following symptoms in the past 7 days?

1. Fever
2. Sore throat
3. Cough
4. Shortness of breath

If the answer is ‘yes’ to any of these questions, proceed to a testing centre for further assessment.

*Source:* Western Cape Department of Health

After testing, people were advised for quarantine (Box 2) until they received the test result. If they were not able to self-quarantine, then a number of facilities such as hotels were available for assisted quarantine.

BOX 2 Advice for self-quarantine while awaiting result.

Self-quarantine:

- If you have been at work, you must stop working and go home.
- You are to stay at home, and must not go out of your place.
- At home, you need to limit physical contact with your family (i.e. no touching, kissing, hugging, etc.).
- If possible, stay in separate rooms, and use separate bathrooms.
- If possible, separate cutlery and crockery.
- Do not allow visitors into your home.
- If you need supplies or groceries, ask someone who is not in quarantine to shop for you and bring to your home.
- If you are on chronic medication, you make a plan for someone to collect medication if it is due.
- Practise good hand hygiene, cough etiquette, clean frequently used surfaces with disinfectant and open windows for ventilation.

*Source:* Western Cape Department of Health

By the end of the third week of CST, the programme had screened 70 251 people and performed 6127 tests, of which 208 were found positive (3.4%), although backlog of tests could increase the number of positive cases.

## Strengths and weaknesses

The pre-existing COPC approach provided essential ingredients for rapidly creating a CST programme. Tackling the epidemic in communities, rather than in hospitals, is a lesson learnt from HIV epidemic.^3^

The failings of CST were both procedural and logistic. Creating a screening tool was difficult when the case definition shifted from week to week in response to the changing nature of the epidemic. This also created some confusion between healthcare workers. It was necessary to amend screening questions to focus on acute illness as many of the symptoms overlapped with that of TB, a highly prevalent infection in Cape Town.

People with moderate or severe COVID-19 were likely to seek medical attention anyway. Creating a tool with sufficient sensitivity for mild cases that did not result in too many false positives (leading to high levels of unnecessary testing and crowding at testing stations) or too many false negatives (leading to cases being missed and continuing to transmit the virus) was a difficult conundrum. The capacity of National Health Laboratory Service in terms of volume of tests and turnaround time was also an issue.

Logistical failings related to staffing, transport, security, consumables, ablution facilities, data collection and capturing, and adequate personal protective equipment (PPE). Data collection was paper-based, with inherent limitations that affected data quality. The Department of Health called for local non-professional volunteers to assist with staffing.

The low positive predictive value of screening questions usually brings the value of screening into question. If only 3% are true positives, then 97% are false positives. In terms of COVID-19, however, 208 undetected cases could become 13 312 new cases within 4 weeks. Community screening and testing also provided awareness and health education on COVID-19 and helped communities to take the epidemic more seriously.

## Opportunities and threats

The value of COPC in implementing CST has highlighted the importance of completing this transformation of the primary health care system. The needs of CST could also leapfrog over some of the pre-existing barriers to COPC, such as access to m-health technology and building integrated one facility with multiple connected teams. The Department of Health is creating a smartphone app for data collection and also providing cell phones.

The most tangible threat to CST is the security situation. Lockdown regulations have led to a lack of income and food insecurity. The CST strategy has compelled teams to operate in the most unstable areas and only intermittent police protection has been available. Teams experienced mugging, witnessed looting and exchange of gunfire, and felt threatened by community members in a number of circumstances. This may also be symptomatic of a more generalised distrust towards the state and its services amongst poor communities. For the teams, this has led to post-traumatic stress, and reduced morale and willingness to participate.

Going forward heightened security measures would be needed from private security companies or even the army. Provision of relief packages to poor communities on humanitarian grounds could mitigate the deteriorating security conditions.

The other significant threat to this CST strategy is the increasing turn-around-time for test results as laboratories are overwhelmed. If cases and contacts cannot be identified quickly then screening and testing becomes ineffective.

## Conclusion

Cape Town has targeted CST around cases in vulnerable communities. This would help to slow down transmission of COVID-19 and enable health services to prepare for the peak of the epidemic. Implementation of CST was made easier by a pre-existing commitment to COPC, and also highlights the importance of COPC principles in transforming PHC. Designing a tool with sufficient predictive value and overcoming logistic issues were key challenges. Safety and security issues as well as laboratory capacity are main threats to CST.
