# Corrigendum: Community-based screening and testing for Coronavirus in Cape Town, South Africa: Short report

**DOI:** 10.4102/phcfm.v13i1.2832

**Published:** 2021-11-15

**Authors:** Neal David, Robert Mash

**Affiliations:** 1 Metropolitan Health Services, Western Cape Department of Health, Cape Town, South Africa; 2 Division of Family Medicine, School of Public Health and Family Medicine, University of Cape Town, Cape Town, South Africa; 3 Division of Family Medicine and Primary Care, Faculty of Medicine and Health Sciences, Stellenbosch University, Cape Town, South Africa

In the version of the article initially published, David N, Mash R. Community-based screening and testing for Coronavirus in Cape Town, South Africa: Short report. Afr J Prm Health Care Fam Med. 2020;12(1), a2499. https://doi.org/10.4102/phcfm.v12i1.2499, one affiliation for the first author, Neal David, was omitted in the ‘Authors’ and ‘Affiliations’ sections. The following affiliation should be added as his second affiliation: Division of Family Medicine, School of Public Health and Family Medicine, University of Cape Town, Cape Town, South Africa.

This correction does not alter the study’s findings of significance or overall interpretation of the study’s results. The authors apologise for any inconvenience caused.

